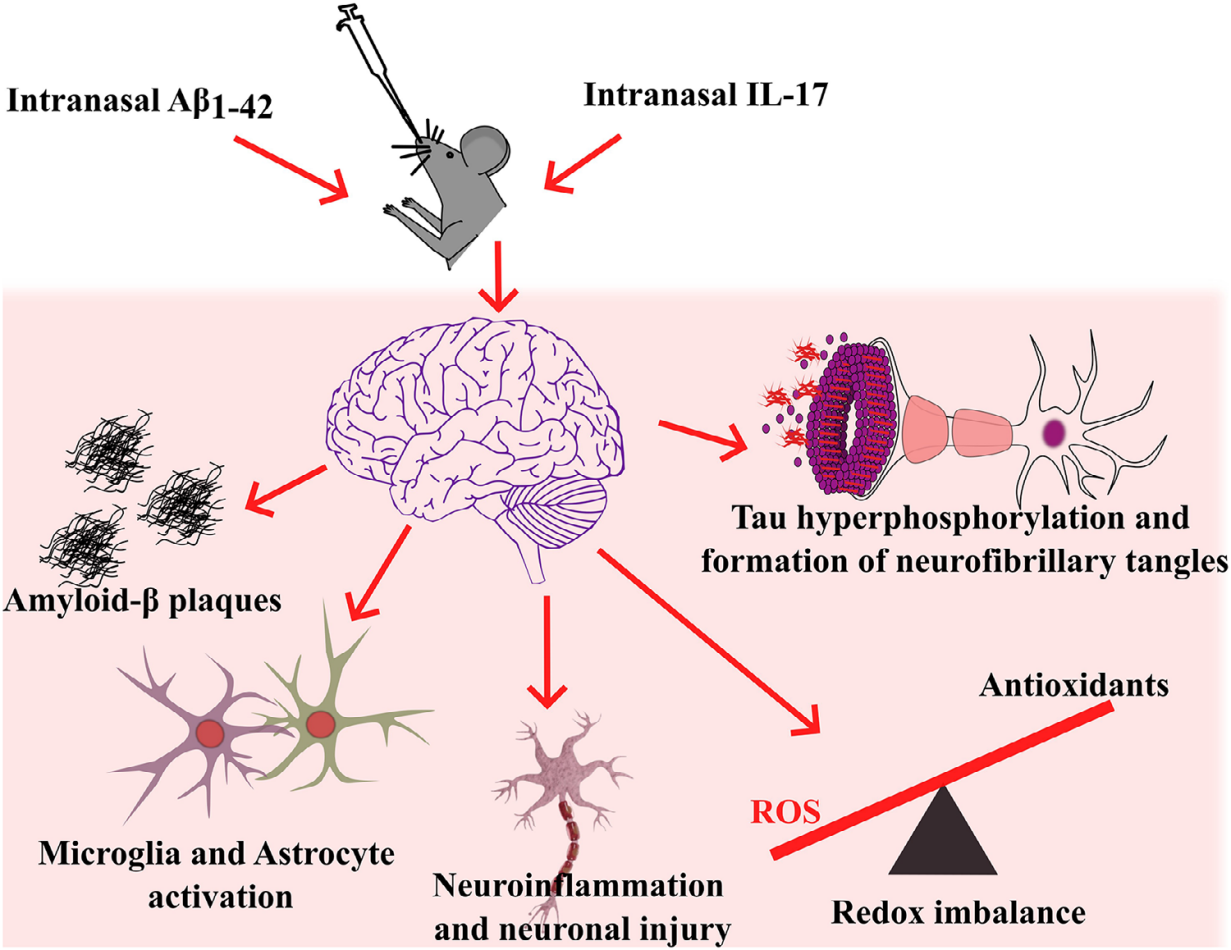# The role of IL‐17A in amplifying neuroinflammation and pathological markers in Alzheimer's disease

**DOI:** 10.1002/alz70855_096840

**Published:** 2025-12-23

**Authors:** Avtar Singh Gautam, Rakesh Kumar Singh

**Affiliations:** ^1^ National Institute of Pharmaceutical Education and Research, Raebareli, Lucknow, Uttar Pradesh, India

## Abstract

**Background:**

Neuroinflammation in Alzheimer's disease (AD) plays a critical role in inducing cellular injury and exacerbating disease pathology. Among the proinflammatory cytokines, interleukin‐17A (IL‐17A) has been significantly associated with AD, amplifying neuroinflammation during disease progression.

**Method:**

This study explored the role of IL‐17A in exacerbating amyloid‐beta (Aβ)‐induced pathology. AD pathology in mice was induced through repeated intranasal administration of Aβ alongside recombinant mouse IL‐17A (rmIL‐17) at doses of 1, 2, and 4 µg/kg for seven alternate days.

**Result:**

While combining rmIL‐17 with Aβ did not severely impact memory when compared to Aβ, it markedly intensified IL‐17A‐mediated signaling. This combination elevated proinflammatory cytokines, increased oxidative stress, and reduced antioxidant levels in the hippocampus and cortex. Interestingly, the co‐administration of rmIL‐17 and Aβ also upregulated key AD structural markers, including pTau, amyloid‐beta, and BACE1, in the brain regions. Furthermore, it activated astrocytes and microglia, leading to a shift in microglial polarization from anti‐inflammatory to pro‐inflammatory states.

**Conclusion:**

These findings highlight the potential of IL‐17A to aggravate AD pathology and emphasize its significance as a therapeutic target for controlling disease progression